# Predictive Equation to Estimate Resting Metabolic Rate in Older Chilean Women

**DOI:** 10.3390/nu14153199

**Published:** 2022-08-04

**Authors:** Eduard Maury-Sintjago, Carmen Muñoz-Mendoza, Alejandra Rodríguez-Fernández, Marcela Ruíz-De la Fuente

**Affiliations:** 1 Department of Nutrition and Public Health, Universidad del Bío-Bío, Chillan 3780000, Chile; 2 Auxology, Bioanthropology, and Ontogeny Research Group (GABO), Faculty of Health and Food Sciences, Universidad del Bío-Bío, Chillan 3780000, Chile; 3 Department of Nursing, Universidad del Bío-Bío, Chillan 3780000, Chile; 4 Research Group on Aging (GIE-UBB), Faculty of Health and Food Sciences, Universidad del Bío-Bío, Chillan 3780000, Chile

**Keywords:** resting metabolic rate, older adult, waist circumference, indirect calorimetry, predictive equations

## Abstract

Resting metabolic rate (RMR) depends on body fat-free mass (FFM) and fat mass (FM), whereas abdominal fat distribution is an aspect that has yet to be adequately studied. The objective of the present study was to analyze the influence of waist circumference (WC) in predicting RMR and propose a specific estimation equation for older Chilean women. This is an analytical cross-sectional study with a sample of 45 women between the ages of 60 and 85 years. Weight, height, body mass index (BMI), and WC were evaluated. RMR was measured by indirect calorimetry (IC) and %FM using the Siri equation. Adequacy (90% to 110%), overestimation (>110%), and underestimation (<90%) of the FAO/WHO/UNU, Harris–Benedict, Mifflin-St Jeor, and Carrasco equations, as well as those of the proposed equation, were evaluated in relation to RMR as measured by IC. Normal distribution was determined according to the Shapiro–Wilk test. The relationship of body composition and WC with RMR IC was analyzed by multiple linear regression analysis. The RMR IC was 1083.6 ± 171.9 kcal/day, which was significantly and positively correlated with FFM, body weight, WC, and FM and inversely correlated with age (*p* < 0.001). Among the investigated equations, our proposed equation showed the best adequacy and lowest overestimation. The predictive formulae that consider WC improve RMR prediction, thus preventing overestimation in older women.

## 1. Introduction

Underestimating or overestimating the energy requirements of an individual leads to inadequate diet prescription and nutritional food management. Energy balance determines body weight based on body mass and the relationship between energy intake and expenditure [1].

The main components of total energy expenditure include dietary thermogenesis, physical activity, and basal metabolic rate (BMR). BMR is defined as the minimum energy expenditure rate that is compatible with life, representing 50% to 75% of the total daily energy expenditure based on the level of physical activity, and it decreases with age at a rate of 1% to 2%, on average, per decade from the second to the seventh decade of life [2].

The difference between resting metabolic rate (RMR) and BMR is <10% [3]. The principal determinant factors of BMR include fat-free mass (FFM), body size, age, sex, and hormone production [4], with FFM as the main determinant [5]. However, body fat also influences BMR according to studies conducted with youth, middle-aged adults [6], and persons with obesity [7,8].

It is also important to note that the metabolic activity of body adipose tissue shows regional variation. Body fat located in the abdominal region (visceral adiposity) has higher metabolic activity than fat located in the gluteal-femoral region [9]. Regarding the relationship between RMR and abdominal fat distribution, research has been inconclusive, as some studies have shown a relationship [10], whereas others have not [11,12].

Furthermore, aging decreases all components of total energy expenditure [13], in addition to significant changes in body composition; older adults show decreased FFM and increased body FM, with a predominantly central distribution [14]. Waist circumference (WC) measurement is a very good predictor of this [15].

The gold standard to determine RMR is indirect calorimetry (IC). Indirect calorimetry calculates RMR by measuring the oxygen and carbon dioxide concentrations found in exhaled air [16]. The IC method is non-invasive and widely validated [17]. However, its application is limited because of its high cost and requirements for both specialized equipment and trained personnel.

Routine clinical practice requires RMR measurement to provide a correct energy prescription. To this end, predictive equations derived from regression models are used, which usually include variables such as sex, age, weight, and height. Such predictive equations are easy to use, cost-free, and always available. Among the most used equations are the Harris–Benedict [18], FAO/WHO/UNU [19], and Mifflin-St Jeor [20] equations. The latter is recommended by the American Dietetic Association (ADA) [21].

A high error rate has been observed when applying predictive equations, mainly associated with overestimates of RMR [22]. It is possible that such errors can be corrected by incorporating other measures. It has been reported that including body composition data and some body circumference measurements, such as WC, improved the estimation of predictive equations [23]. This is particularly relevant for patients with obesity and older adults [24,25].

Therefore, the objective of the present study was to analyze the influence of waist circumference in predicting resting metabolic rate and propose a specific estimation equation for older Chilean women.

## 2. Materials and Methods

### 2.1. Study Design

This was an analytical cross-sectional study.

### 2.2. Sample

The sample was non-probabilistic and consisted of 45 older women between the ages of 60 and 85 years, who were recruited at a primary healthcare center in south-central Chile. All participants met the following selection criteria: general good health, absence of hypermetabolic disease, stable body weight (±3 kg) in the last 6 months, and absence of medication consumption that modifies RMR prior to its measurement.

The present study was reviewed and approved by the Ethics and Biosecurity Committee of the Universidad del Bío-Bío, and all participants provided informed consent. The procedures were conducted according to the ethical norms of the Declaration of Helsinki and the Council for International Organizations of Medical Sciences (CIOMS).

### 2.3. Anthropometric Measurements

Weight and height were categorized using cutoff points established by the WHO in 1998 [26]. Body weight was measured using a balance (SECA, model 713) with a scale from 2 to 130 kg and 0.2 kg precision. A measuring rod (SECA, model 212) with a minimum graduation of 1 mm was used to obtain body height. Skinfolds (biceps, triceps, subscapular, and suprailiac) were measured with a Lange caliper (Beta Technology Incorporated, Cambridge, MD, USA) with a 1 mm sensitivity and 0–67 mm graduation. All measurements were taken in triplicate [27,28,29]. Body mass index (BMI) was used to classify the nutritional state, with cutoff points suggested for persons over 50 years of age [30]. WC was measured with a non-elastic metric measuring tape (SECA, model 201) during the vacuum between the end of expiration and the beginning of normal inspiration, midway between the left costal margin (lower edge of the tenth rib) and the iliac crest (anterior-superior iliac spine). WC measurement was considered an indicator of abdominal fat distribution; the cutoff point for normality was <80 cm, whereas values ≥ 80 cm were categorized as abdominal fat distribution [26].

### 2.4. Body Composition

%FM was calculated according to the Siri equation [31], expressed as:

Percentage body fat = [(4.95/density) − 4.50] × 100

Body density (D) was obtained with the equation proposed by Durnin and Womersley and expressed as:

D = C − M × log10 ∑ 4-site skinfolds

where C and M are the sum of the four skinfolds (biceps, triceps, subscapular, and suprailiac), using the C and M coefficients of the sum of the four skinfolds that are specific to each age group for the female sex shown in the tables developed by Durnin and Womersley [32]. %FFM was then obtained by calculating the difference between %FM and 100%.

### 2.5. Indirect Calorimetry (IC)

The IC method was used to obtain RMR. Participants were asked not to consume caffeine, smoke, or exercise for 24 h and to fast for 10 to 12 h prior to the measurement. We verified that the subjects had no previous history of thyroid disease or anemia. Vital signs were monitored during the procedure: axillary body temperature < 37 °C and respiratory rate between 12 and 18 breaths per minute. Measurements were taken early in the morning.

Prior to measurement, the equipment was calibrated according to the technical manufacturing specifications and using standard gases. A CO_2_ concentration <5% and an ambient temperature between 20 and 24 °C were provided in the environment. Following a 30 min rest, a canopy was placed over the participant’s head, and expired air was measured with a respiratory analyzer (VMAX 29 N, SensorMedics Corp., Yorba Linda, CA, USA). A metabolic chart reading was taken once the stable period was reached, which included VO_2_ consumption, VCO_2_ production, and the respiratory quotient (RQ). Test validity was confirmed by the RQ value obtained from the eliminated VCO_2_ and consumed VO_2_, with a normal physiological range from 0.7 to 1.0, and by verifying the fluctuation in the VCO_2_ (mL/min) and VO_2_ (mL/min) exchange [33]. Steady state was defined as the first 5 min period with a coefficient of variation (CV) ≤ 10% for both VO_2_ and VCO_2_ [34].

### 2.6. Adequacy of Predictive Equations with Resting Metabolic Rate Measured by Indirect Calorimetry (RMR IC)

The adequacy of the FAO/WHO/UNU 1985 [19], Harris–Benedict 1919 [18], Mifflin-St Jeor 1990 [20], and Carrasco [35] predictive equations, as well as that of the equation proposed in the present study, was determined with respect to the RMR IC. Adequacy was defined as a percentage difference between the estimated RMR and the RMR IC within ±10%; when the difference was <90% or >110%, the result was categorized as underestimation or overestimation, respectively [36]. The percentage difference between estimated RMR and RMR IC was calculated for each woman in the study using the following equation: (RMR_estimated_ − RMR IC)/RMR IC × 100.

### 2.7. Statistical Analysis

The quantitative variables were described with central tendency and dispersion measurements, and their normal distribution was verified by the Shapiro–Wilk test. Pearson’s correlation analysis was used to evaluate the correlation between RMR IC, anthropometric measurements, and age. Pearson’s correlation analysis was also used for the adjusted RMR IC to control its variability due to body composition of FFM, which was based on a study by Ravussin and Bogardus [37] and expressed as RMR IC_adjusted_ = RMR IC_mean_ + RMR IC_individual_ − RMR_predicted_. The predicted metabolic rate is the calculated rate obtained by using FFM in the linear equation generated in the studied sample.

The prediction of RMR IC according to body composition, age, and WC was modeled by multiple linear regression analysis. Assumptions of collinearity through the variance inflation factor (VIF), error normality (Shapiro–Wilk), and homogeneity of variance (Breusch–Pagan) were verified.

The mean estimated RMR of the sample derived from the analyzed predictive equations was compared with the mean RMR IC of the sample according to Student’s *t*-test. Regression equations with probability value (*p*), multiple coefficient of determination (R^2^), and standard error of estimate (SEE) were obtained.

We analyzed the consistency between the RMR estimated by the proposed equation and the RMR IC according to the Bland and Altman method [38], as well as the intraclass correlation coefficient (ICC).

The normal distribution was verified by the Shapiro–Wilk test. RMR values obtained using the investigated equations were compared using ANOVA and Tukey’s test. The analyses were performed with the STATA 14.0 software (StataCorp LP, College Station, TX, USA) at α = 0.05 level of significance.

## 3. Results

Table 1 shows the characteristics of the participants. The group under study consisted of 45 women between the ages of 60 and 85 years with a mean age of 66.0 ± 3.8. Other variables were: height, 1.53 ± 0.1 m; body weight, 66.2 ± 11.2 kg; WC, 90.0 ± 11.1 cm; and BMI, 28.3 ± 4.3. The WC was ≥80 cm in 82.2% of the sample, showing abdominal fat distribution. According to BMI, 68.9% of participants had a normal nutritional status, 6.6% were suffered undernutrition, and suffered 24.4% overnutrition. Mean RMR IC was 1083.6 ± 171.9 kcal/day. Other general characteristics are described in Table 1.

RMR IC was significantly and positively correlated with FFM (*r* = 0.70), weight (*r* = 0.64), WC (*r* = 0.56), FM (*r* = 0.51), and BMI (*r* = 0.40) and inversely correlated with age (*r* = −0.48). However, RMR IC adjusted for FFM only showed a statistically significant and inverse correlation with age (*r* = −0.42) (*p* < 0.0001) (data not shown).

Table 2 shows that most of the variance in RMR is explained by FFM (*R*^2^ = 0.59). WC explained an additional 6% of the variance in the estimated RMR (*R*^2^ = 0.65). Stepwise multiple linear regression analysis showed that age, FFM, FM, and WC were significant predictive variables explaining RMR in the group under study. The predictive equation was RMR = 1012 − 17.6 AGE (year) − 11.8 FM (kg) + 23 FFM (kg) + 8.1 WC (cm).

**Figure 1:**
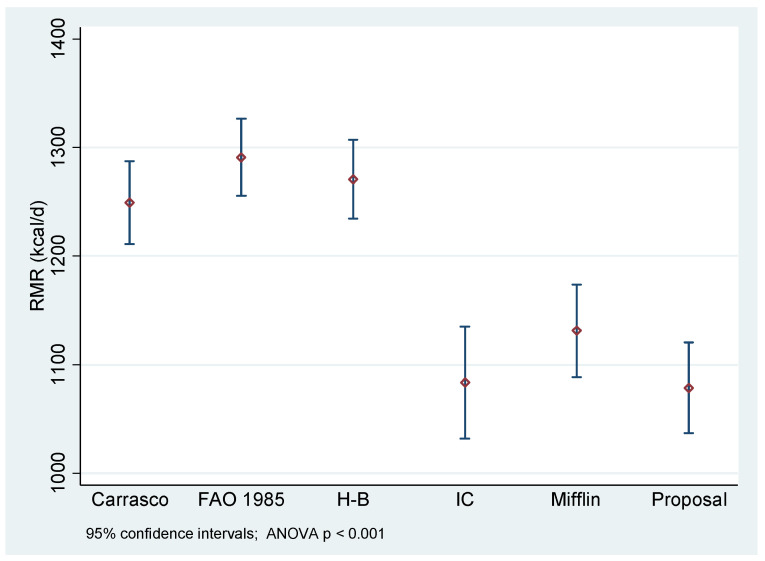
Resting metabolic rate measured by indirect calorimetry versus estimation by predictive equations in older Chilean women. H-B: Harris–Benedict equation; Mifflin: Mifflin-St Jeor equation; IC: indirect calorimetry.

Figure 1 shows the statistically significant differences in mean RMR according to the estimation equation. The measurement of RMR IC is statistically different from RMR estimated by the Carrasco, FAO/WHO/UNU 1985, and Harris–Benedict equations (*p* < 0.01). However, there were no statistically significant differences in the measurement of RMR IC as related to estimation with the Mifflin-St Jeor equation and the equation proposed in the present study (*p* > 0.05).

Table 3 shows the percent of adequacy of the predictive equations versus the RMR IC. In 80% of the women under study, the proposed equation showed adequacy between 90% and 110% for RMR IC, which was the best adequacy among the analyzed equations. In only 11% of the women, this equation estimated a value >110% of that determined by the RMR IC; it also had the lowest overestimation (11%) among all analyzed equations.

**Figure 2:**
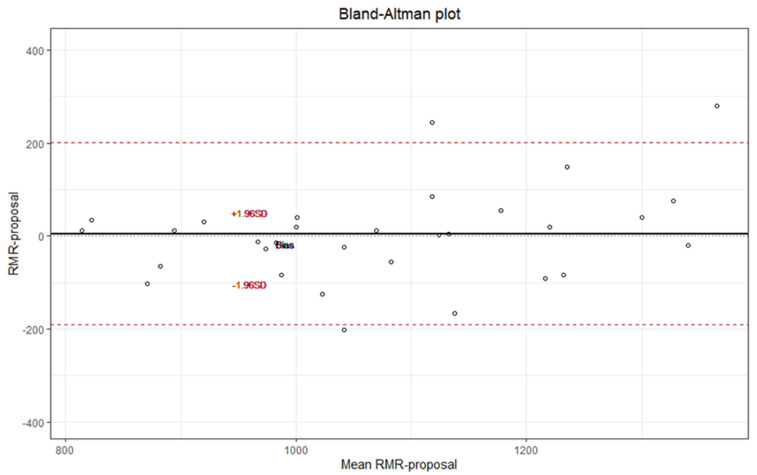
Consistency between the RMR estimated by the proposed equation versus the RMR IC according to the Bland and Altman method.

Figure 2 shows that the proposed equation, which includes age, FM, FFM, and WC variables, concurs with the RMR IC according to the Bland and Altman method. The intraclass correlation coefficient (ICC) with ICC = 0.798 (95% CI 0.661–0.883) exhibited good concordance.

## 4. Discussion

The main determinant of the RMR IC in the studied women was FFM, which is consistent with findings reported in other studies [4,5]. Other determinants, in decreasing order, were body weight, WC, and body fat.

There was a positive and significant correlation between body weight and RMR IC (*r* = 0.64), which was explained by the observed proportionality of the FM and FFM components, as well as their influence on each other. However, as aging progresses, RMR/kg of body weight decreases, which is not only explained by changes in body composition [7,15,39].

WC represents a measurement of adiposity related to abdominal fat accumulation [15]. Abdominal fat is characterized as a metabolically active tissue with increased blood flow, norepinephrine responsiveness, sympathetic nervous system activity, lipolysis rate, and low sensitivity to the antilipolytic effect of insulin compared with subcutaneous adipose tissue [10,40,41]. It is also associated with increased metabolic risk of hyperglycemia, glucose intolerance, hypertension, and insulin resistance [42,43,44,45]. Older women were reported to have a mean WC of 90 cm; this value is higher than the established normal WC of <80 cm [26,46]. This is consistent with a predominantly central fat distribution described in older adults [47].

We observed a statistically significant and positive correlation between WC and RMR IC (*p* < 0.0001), which is similar to findings reported in other studies [10,48]. This confirms our proposed hypothesis of a positive correlation between abdominal fat distribution and RMR IC. Furthermore, multiple linear regression analysis revealed that including WC contributed to prediction of RMR beyond body composition, with an additional 6% in the variation of the estimated RMR of the sample. This was similar to results reported by Lührmann et al. [10] and those reported in study conducted with obese and non-obese premenopausal women [49]. Some studies have included WC among the variables in predictive equations to determine RMR [50,51].

Although body FM was also positively correlated (*r* = 0.51) with RMR IC, it was to a lesser degree than WC (*r* = 0.56) and did not influence the variation in the estimated RMR according to multiple regression analysis. This contrasts with findings of another study in which body fat accounted for between 1% and 10% of the variance in RMR in young and middle-aged women [7].

Aging entails reduced total energy expenditure associated with a decrease in each of its components, that is, RMR, thermogenic effect, and physical activity [12]. In our study, RMR IC adjusted for FFM to control for the variation attributable to differences in body composition only showed a statistically significant and inverse correlation with age (*p* < 0.0001). This phenomenon is attributed to decreased FFM and the associated increased body FM that occurs with age, as well as to body fluid content, alterations in body temperature and hormones, total body area, physical inactivity, and genetic factors [52].

The estimation of BMR with predictive equations derived from studies conducted in populations with racial/ethnic characteristics different from those found in Chile, such as the FAO/WHO 1985 and Harris–Benedict equations, showed statistically significant differences with the mean RMR IC of the group under study (*p* > 0.05), which is in agreement with previously reported findings [53]. In addition, both equations overestimated RMR IC in 80% of the women in the study. Overestimating energy requirements can lead to inadequate calorie recommendations and promote unwanted weight gain in the medium-to-long term.

The equation proposed by Carrasco [35] includes body weight and age data of each subject and was derived from a study conducted with Chilean women between the ages of 18 and 74 with normal nutritional status, overweight, and obesity. However, the estimation of RMR in the group also showed statistically significant differences for RMR IC and an important overestimation in 71% of the women in the study.

Unlike the aforementioned equations, use of the Mifflin-St Jeor equation and our proposed equation to estimate the RMR of women showed no statistically significant differences with the RMR IC (*p* > 0.05). The Mifflin-St Jeor equation is recommended by the ADA because it has good adequacy in subjects with normal weight and obesity. However, it has also been described as producing a substantial clinical error of approximately 20% overestimation and underestimation of RMR; in addition, the racial origin of the population from which it was derived is unknown [20]. The proposed equation derived from our study, which considered age (year), FFM (kg), body mass (kg), and WC (cm) data, was the best predictive equation among all analyzed equations in 80% of the women, resulting in the lowest overestimation in only 11%. Our proposed equation also showed good consistency according to the Bland and Altman method with extreme differences <200 kcal, which is lower than those estimated by predictive equations. The ICC 0.798 (95% CI: 0.661–0.883) also showed good concordance.

The strength of our study is that we carefully controlled for the effect of several potentially important confounding factors, such as the absence of medical conditions that affect the RMR of the older women and the possible thermogenic effects of food, caffeine, and nicotine.

Estimation of RMR using traditional predictive equations is not error-free and can lead to inaccurate estimation when calculating the energy requirements of a subject, especially if they differ in terms of ethnic/racial characteristics from the population from which they are derived.

The proposed equation showed that considering body composition data, especially WC, improved the prediction of RMR, which is consistent with previously reported results [54]. Therefore, its use could be a clinically applicable option for estimating RMR in older Chilean women when it is not possible to take a measurement by the indirect calorimetry method. However, further studies are required to validate its use in the Chilean population.

## 5. Conclusions

In conclusion, including body composition, especially waist circumference, as an indicator of abdominal fat distribution improves the prediction of RMR with the proposed equation for the group of older women under study. This reduces errors, mainly due to overestimation of energy requirements and their implications on body weight.

## Figures and Tables

**Table 1:** Age, anthropometry, body composition, and resting metabolic rate of the participants measured by indirect calorimetry.

Variable	Women (*n* = 45) Mean ± SD
Age (years)	66.0 ± 3.8
Weight (kg)	66.2 ± 11.2
Height (m)	1.5 ± 0.1
BMI (kg/m^2^)	28.3 ± 4.3
AC (cm)	32.3 ± 4.2
BSF (mm)	16.7 ± 9.1
TSF (mm)	21.0 ± 8.9
SSF/(mm)	24.8 ± 10.8
SBSF (mm)	23.8 ± 9.5
Sum of skinfolds (mm)	118.5 ± 38.7
FM (kg)	29.6 ± 7.5
FFM (Kg)	36.6 ± 4.7
WC (cm)	90.0 ± 11.1
RMR IC (kcal/day)	1083.6 ± 171.9

SD: standard deviation; BMI: body mass index; AC: arm circumference; BSF: bicep skinfold; TSF: tricep skinfold; SSF: suprailiac skinfold; SBSF: subscapular skinfold; FM: fat mass; FFM: fat-free mass; WC: waist circumference; RMR IC: resting metabolic rate measured by indirect calorimetry.

**Table 2:** Predictive equations used to estimate resting metabolic rate based on fat-free mass, fat mass, age, and waist circumference.

Regression Equation	*p*	*R* ^2^	SEE	β
RMR = 1505.3 − 16.4 AGE + 7.3 WC	<0.01	0.44	131.7	(−0.37; 0.47)
RMR = 1808.6 − 14.9 AGE + 8.7 FM	<0.01	0.35	141.2	(−0.33; 0.38)
RMR = 1199.0 − 14.4 AGE + 22.8 FFM	<0.01	0.59	112.4	(−0.32; 0.62)
RMR = 1231.6 − 15.0 AGE − 1.4 FM + 24.2 FFM	<0.01	0.59	113.1	(−0.34; −0.07; 0.66)
RMR = 1012.0 − 17.6 AGE − 11.8 FM + 23.0 FFM + 8.1 WC	<0.01	0.65	105.5	(−0.39; −0.52; 0.62; 0.53)

*R*^2^: coefficient of determination; SEE: standard error of estimate; RMR: resting metabolic rate; WC: waist circumference; FM: fat mass: FFM: fat-free mass.

**Table 3:** Percent adequacy (overestimation/underestimation) of predictive equations as related to resting metabolic rate measured by indirect calorimetry in the studied sample.

Predictive Equation	Underestimation (<90%)	Adequacy (90% to 110%)	Overestimation (>110%)
Proposal	8.8	80	11
Mifflin-St Jeor	8.8	60	41
Harris–Benedict	0	20	80
FAO/WHO/UNU	0	20	80
Carrasco	4.4	24	71

## Data Availability

The data presented in this study are available on request from the corresponding authors.
